# Salivary microRNA and Metabolic Profiles in a Mouse Model of Subchronic and Mild Social Defeat Stress

**DOI:** 10.3390/ijms232214479

**Published:** 2022-11-21

**Authors:** Yuta Yoshida, Yuhei Yajima, Kina Kawakami, Shin-ichi Nakamura, Takamitsu Tsukahara, Katsutaka Oishi, Atsushi Toyoda

**Affiliations:** 1 Department of Food and Life Sciences, College of Agriculture, Ibaraki University, Mito 300-0393, Japan; 2 United Graduate School of Agricultural Science, Tokyo University of Agriculture and Technology, Tokyo 183-8509, Japan; 3 Kyoto Institute of Nutrition & Pathology, Kyoto 610-0231, Japan; 4 Healthy Food Science Research Group, Cellular and Molecular Biotechnology Research Institute, National Institute of Advanced Industrial Science and Technology, Tsukuba 305-8566, Japan; 5 Department of Applied Biological Science, Graduate School of Science and Technology, Tokyo University of Science, Noda 278-8510, Japan; 6 Department of Computational Biology and Medical Sciences, Graduate School of Frontier Sciences, The University of Tokyo, Kashiwa 277-0882, Japan; 7 School of Integrative and Global Majors, University of Tsukuba, Tsukuba 305-8577, Japan

**Keywords:** heart, metabolite, microRNA, saliva, social defeat stress

## Abstract

Identification of early biomarkers of stress is important for preventing mood and anxiety disorders. Saliva is an easy-to-collect and non-invasive diagnostic target. The aim of this study was to characterize the changes in salivary whole microRNAs (miRNAs) and metabolites in mice subjected to subchronic and mild social defeat stress (sCSDS). In this study, we identified seven upregulated and one downregulated miRNAs/PIWI-interacting RNA (piRNA) in the saliva of sCSDS mice. One of them, miR-208b-3p, which is reported as a reliable marker for myocardial infarction, was upregulated in the saliva of sCSDS mice. Histological analysis showed frequent myocardial interstitial fibrosis in the heart of such mice. In addition, gene ontology and pathway analyses suggested that the pathways related to energy metabolism, such as the oxidative phosphorylation and the pentose phosphate pathway, were significantly related to the miRNAs affected by sCSDS in saliva. In contrast, salivary metabolites were not significantly changed in the sCSDS mice, which is consistent with our previous metabolomic study on the plasma of sCSDS mice. Taken in the light of previous studies, the present study provides novel potential stress biomarkers for future diagnosis using saliva.

## 1. Introduction

Depression is one of the most frequent disorders, affecting more than 264 million people worldwide [1]. Depression not only severely impairs a patient’s quality of life due to a depressed mood, anxiety, and loss of pleasure but can also be a contributing factor for various diseases [2]. Selective serotonin reuptake inhibitors are the most widely used antidepressants for patients with major depressive disorder (MDD). However, it has been suggested that approximately 30% of patients show resistance to treatment with selective serotonin reuptake inhibitors [3]. Therefore, prevention and early diagnosis of depression are essential and, consequently, it is important to identify diagnostic biomarkers of psychological stress. Saliva is an easy-to-collect, non-invasive, and cost-effective diagnostic target. Saliva collection does not require highly trained professionals, and salivary diagnosis is highly practical and free from infection risk through contaminated needles [4]. In fact, because salivary cortisol responds to stress conditions [5], it is reasonable to focus on the salivary biomarkers of depression. Thus, the present study focused on salivary biomarkers for the early diagnosis of stress.

Depression is generally diagnosed using subjective methods, such as interview-based diagnoses [6], suggesting that the objective biomarkers for stress and depression could help improve the diagnosis of depression. Therefore, multiple studies to identify the early biomarkers of stress and depression have been performed [7,8,9,10,11,12,13,14,15,16,17,18]. Previously, microRNA (miRNA) profiles were investigated in patients with MDD in whole blood [7,8], blood serum [9,10], peripheral blood mononucleocytes [10,11,12], and the prefrontal cortex [8,14,15,16]. It was suggested that miRNA profiles are significantly altered in patients with MDD. In addition, metabolomic analyses were performed using plasma samples from patients [17]. However, the salivary biomarkers of stress and depression were not examined. Salivary stress biomarkers enable the early detection of depression for those who hesitate to use the mental health services [18], because saliva collection can be performed at home without any clinical equipment. In particular, we focused on miRNAs as potential biomarkers, because miRNAs were consistently present in the salivary exosomes, which protect miRNAs from digestion [4]. In this study, we investigated salivary miRNAs and metabolic profiles in an animal model of depression, which fulfills predictive validity [19,20], in advance of future studies using human saliva samples. In addition, as studies on animal models have the advantage that the genetic and environmental backgrounds of the animals are highly controlled, in contrast with the human studies, we speculated that an animal model could be suitable for the identification of the biomarkers specifically reflected from the pathophysiology of stress. Interestingly, a recent study compared the metabolomic data of patients with MDD and those of mice subjected to chronic social defeat stress (CSDS), and stated that some metabolites were commonly regulated in the plasma of humans and mice [21]. CSDS is widely used as an animal model of depression [19,20]. We have previously established a mouse model of subchronic and mild social defeat stress (sCSDS), and found significant increases in body weight, food intake, water intake, and social avoidance behavior [22,23]. In particular, our sCSDS paradigm consists of a half-scale physical stress condition, compared with the standard CSDS method [24]. Thus, our sCSDS conditions would likely reduce the effects of physical stress and wounds and induce milder phenotypes compared to standard CSDS mice [22]. Therefore, we investigated entire salivary miRNAs and metabolic profiles in sCSDS mice.

## 2. Results

### 2.1. Body Weight, Food Intake, and Water Intake

**Figure 1:**
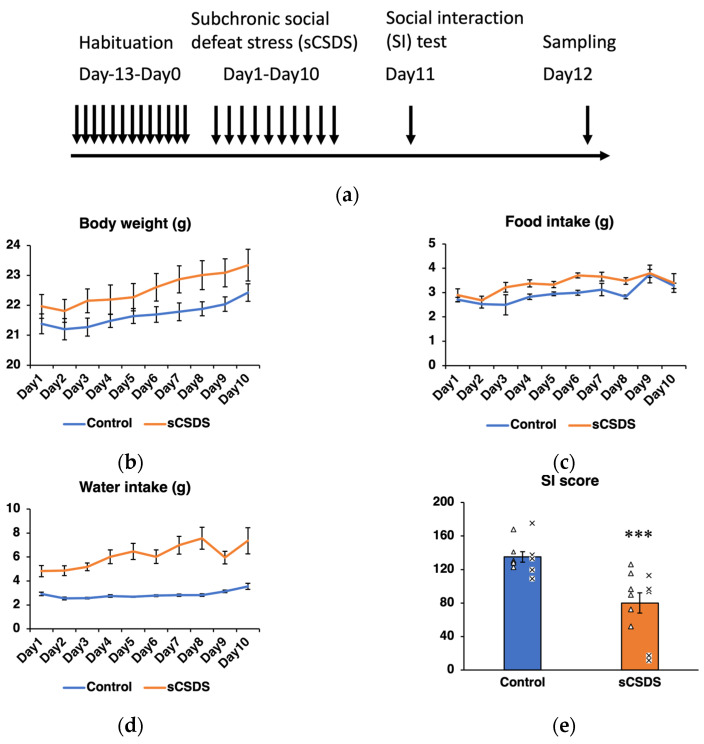
(**a**) Experimental design of the subchronic and mild social defeat stress (sCSDS) paradigm, social interaction (SI) test, and sampling. (**b**–**d**) The changes in the body weight, food intake, and water intake in the sCSDS mice are presented (*n* = 11). Values are expressed as means ± SE (*n* = 11). (**e**) The SI scores of the sCSDS and control mice are presented. The SI scores (% of target absent) are calculated as (interaction time with target present/interaction time with target absent) × 100. The SI scores significantly decreased in the sCSDS mice compared to the control mice by unpaired *t*-test (*** *p* < 0.001, degree of freedom (*df*); 20). Values are expressed as means ± SE (*n* = 11). The individual SI scores of the mice used in the miRNA-seq analysis are presented as cross marks (×; *n* = 6), and those of the mice used in the metabolomic analysis are presented as triangles (△; *n* = 5).

Daily body weight, food intake and water intake were monitored to evaluate the effect of sCSDS (Figure 1b–d). The significant effects of sCSDS on food intake (*p* < 0.001) and water intake (*p* < 0.001) were observed using two-way analysis of variance (ANOVA) (Table 1).

### 2.2. Social Interaction (SI) Test

The SI scores significantly decreased in the sCSDS group (*p* < 0.001; Figure 1e). In the present study, B6 mice in the sCSDS group were selected according to their lower SI scores in advance of subsequent microRNA-seq and metabolome analyses.

### 2.3. Saliva microRNA-seq Analysis

**Figure 2:**
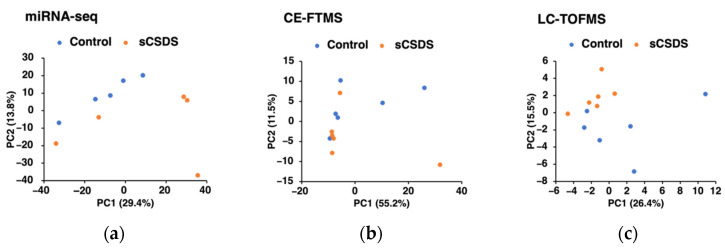
(**a**–**c**) Principal component analysis (PCA) results of control and sCSDS mice used in the miRNA-seq analysis (*n* = 5 in each group); (**a**), capillary electrophoresis Fourier transform mass spectrometry (CE-FTMS) analysis (*n* = 6 in each group); (**b**), and liquid chromatography time-of-flight mass spectrometry (LC-TOFMS) analysis (*n* = 6 in each group) (**c**) are presented.

We identified eight miRNAs/piRNAs, which tended to be up/downregulated in the saliva of sCSDS mice (*q* < 0.1; Table 2; Supplementary Tables S1 and S2). miR-208b-3p was one of the most upregulated miRNAs in the sCSDS group (Table 2). The results of principal component analysis (PCA) of saliva miRNA-seq data are presented in Figure 2a. Interestingly, the miRNA profiles of the control and sCSDS mice were not clearly differentiated by the PC1 and PC2. In addition, the gene ontology term and pathway analyses revealed that several pathways, such as the oxidative phosphorylation and the pentose phosphate pathway, were significantly related to the miRNAs modulated by sCSDS in saliva (Table 3; Supplementary Table S3).

### 2.4. Saliva Metabolome Analyses

In the saliva, 384 and 61 metabolites were detected using capillary electrophoresis (CE)-Fourier transform mass spectrometry (FTMS) and LC-TOFMS analyses, respectively. We found that no metabolites were significantly modified in the saliva of sCSDS mice (*q* > 0.1, Supplementary Tables S4 and S5). The results of the PCA of the saliva metabolome are shown in Figure 2b (CE-FTMS analysis) and 2c (LC-TOFMS analysis); we discovered that the saliva metabolome profiles of the control and sCSDS mice were not clearly differentiated by the PC1 and PC2.

### 2.5. Pathological Analysis of the Heart of sCSDS Mice

**Figure 3:**
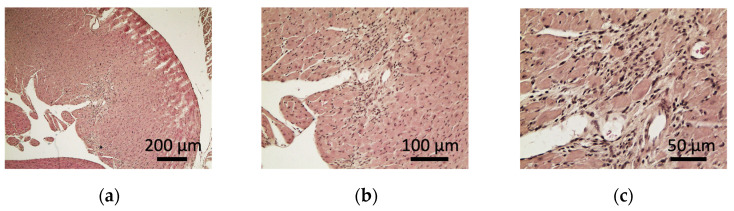

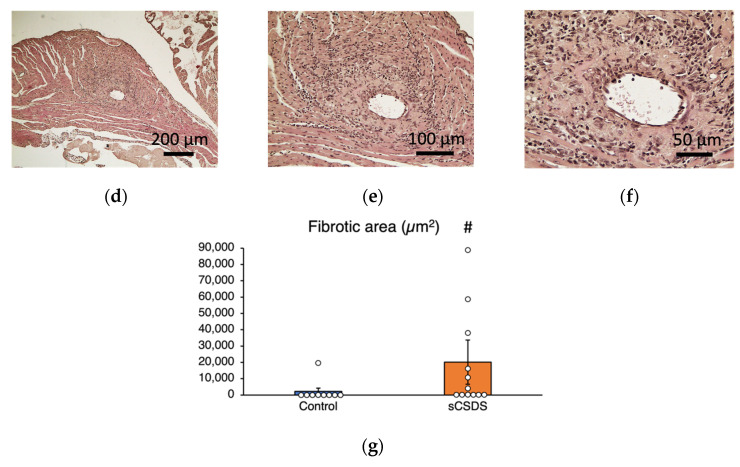
(**a**–**c**) The histological sections of the heart from sCSDS mouse #1 are shown. The fibrotic tissue accumulation is depicted at lower (**a**), middle (**b**), and higher (**c**) magnifications. (**d**–**f**) The histological sections of the hearts from the sCSDS #3 are presented (**a**–**c**). The inflammatory cell infiltration is depicted at lower (**a**), middle (**b**), and higher (**c**) magnifications. (**g**) The average fibrotic area (µm^2^) from 10 continuous sections for each sample is presented in control (*n* = 10) and sCSDS mice (*n* = 13). Values are expressed as means ± SE. # *p* = 0.07 by the unpaired *t*-test (*df*; 21).

Among the ten control mice, the hearts of the eight control mice (control#1, #2, #4, #6, #7, #8, #9, and #10) had no pathological abnormality, and hearts of the two control mice (control#3 and #5) showed myocardial interstitial fibrosis (Table 4; Supplementary Figure S1). However, among the 13 sCSDS mice, the hearts of eight sCSDS mice (sCSDS#1, #2, #3, #4, #5, #7, #8, and #13) showed myocardial interstitial fibrosis, and the hearts of two sCSDS mouse (sCSDS#3 and #9) showed inflammatory cell accumulation, including neutrophils and macrophages (Figure 3a–f; Table 4; Supplementary Figure S1). The hearts of four sCSDS mice (sCSDS #6, #10, #11, and #12) showed no pathological observations (Table 4; Supplementary Figure S1). The average fibrotic areas (µm^2^) of the hearts of sCSDS mice tended to be larger than those of the hearts of control mice (*p* = 0.07; Figure 3).

## 3. Discussion

In the present study, mice subjected to sCSDS showed increased food and water intake compared to the control mice, as previously described [22]. The robust increase in water intake could be related to polydipsia-like behavior [22]. The sCSDS mice also exhibited social avoidance behaviors. Taken together, these results indicate that the mice used in the present study successfully reflected the effects of sCSDS.

Furthermore, in this study, we identified seven upregulated and one downregulated miRNAs/piRNA in the saliva of sCSDS mice. Interestingly, miR-208b-3p, which is downregulated in the nucleus accumbens of CSDS mice [25], was upregulated in the saliva in this study. Thus, it is possible that miR-208b-3p is reversibly modulated in the saliva and nucleus accumbens. The previous studies investigated the miRNAs of patients with MDD, using their whole blood [7,8], blood serum [9,10], peripheral blood mononucleocytes [11,12,13], and prefrontal cortex [8,14,15,16]. In addition, salivary miRNA biomarkers in humans subjected to acute psychological stress using the Trier Social Stress Test were investigated [26]. However, no identical biomarkers were found among these reports and the eight miRNAs identified in the present study. Therefore, the present study identified novel potential candidates of salivary biomarkers reflecting chronic and mild social stress conditions. The present miRNA signals were not identified before and could be novel; however, they could also be a false negative. It is possible that animal models may provide an approach for identifying new potential biomarkers for psychological stress. In the future, investigation of the eight salivary miRNAs/piRNAs, identified in the present study, in humans with chronic and mild psychological stress, will be useful for identifying human salivary stress biomarkers.

The present study found that the expression of miR-208b-3p tended to be upregulated in the saliva of sCSDS mice (Table 2). In particular, miR-208b is expressed in cardiac and skeletal muscles and regulates slow myosin expression in skeletal muscles [27,28,29]. Moreover, miR-208b has also been suggested as a reliable biomarker, which is highly expressed in patients with acute myocardial infarction [30,31], suggesting its role in cardiac systems. Psychological stress is considered an independent risk factor for coronary artery disease and myocardial stunning [32,33]. In addition, mice subjected to social defeat stress showed altered heart rate and cardiac arrhythmias [34,35], while those subjected to two weeks of CSDS showed fibrotic tissue accumulation in the heart [35,36]. Our additional results also indicated that the hearts of sCSDS mice exhibited myocardial interstitial fibrosis at a higher rate compared to the control mice (Figure 3). Thus, it is suggested that upregulation of salivary miR-208b-3p is related to cardiac dysfunction in sCSDS mice. Thus, further studies of myocardial system dysfunction in sCSDS mice will help understand the role of miR-208b-3p, especially studies of psychological stress-induced heart fibrosis by the manipulation of miR-208b-3p expression. The present study also raised the possibility that salivary miR-208b-3p could be a potential biomarker for cardiac disease, in addition to psychological stress. However, we found that miR-208b-3p expression tended to be upregulated in the saliva of sCSDS mice (*q* < 0.1). Thus, further expression analyses in several tissues, including salivary glands and hearts, are required to validate the expression of this miRNA as a reliable biomarker.

In the present study, miR-378a-5p expression was upregulated in mice subjected to sCSDS (Table 2). miR-378a-5p expression is also upregulated in the peripheral blood of patients with bipolar disorder [7]. It has been reported that the knockout of miR-378a causes resistance to high-fat diet-induced obesity by regulating mitochondrial metabolism [37]; thus, miR-378a is considered a potential target for obesity and metabolic syndrome. Bipolar disorder has been suggested to be associated with mitochondrial dysfunction [38]. miR-378 is highly expressed in brown adipose tissue (BAT) [37], and the expression of miR-378 reportedly increased during BAT differentiation [39]. The SDS rats showed BAT thermogenesis and hyperthermia [40], while CSDS mice showed increased BAT weight [41]. Thus, it is possible that BAT could be a potential candidate for salivary miR-378a-5p in sCSDS mice.

We discovered that miR-3064-3p expression was upregulated in the saliva of sCSDS mice (Table 2). It has been reported that miR-3064-3p is involved in cementoblast differentiation [42]. To the best of our knowledge, among the seven upregulated and one downregulated miRNA/piRNAs in the saliva of sCSDS mice, the possible functions of the other miRNA/piRNAs other than miR-208b-3p, miR-378a-5p and miR-3064-3p, have not been reported.

Pathway analyses revealed that multiple pathways were significantly related to the miRNAs up/downregulated by sCSDS (Table 3). The pathways related to energy metabolism, such as oxidative phosphorylation and pentose phosphate pathway, were significantly related to sCSDS (Table 3). In the oxidative phosphorylation pathway, the present gene ontology term analysis suggested that a lipid-soluble part of ATP synthase, *atp5j*, and nicotinamide adenine dinucleotide dehydrogenases, *ndufa8* and *ndufb6*, are related to the miRNAs modulated by sCSDS. It has been suggested that multiple nduf proteins, including ndufa8 and ndufb6, were downregulated by corticosterone treatment in the neural stem cell line, C17.2 cells [43]. In addition, it has been suggested that CSDS downregulates oxidative metabolism in the brain of rats [44]. Moreover, consistent with the present results, multiple studies have commonly suggested that the gut microbiota found in patients with MDD are related to the pentose phosphate pathway [45,46,47]. It is known that the pentose cycle is a major source of nicotinamide adenine dinucleotide phosphate, and this can inactivate reactive oxygen species, which mediates oxidative stress [48]. Multiple studies have suggested that oxidative stress is associated with the pathophysiology of social stress [49] and depression [50]. Therefore, the present study suggested the possibility that miRNAs related to the pentose phosphate pathway might play important roles in oxidative stress responses in depression. Taken together, the present results may provide new insights into the involvement of miRNAs in energy metabolism induced by sCSDS.

Recently, it was reported that miRNAs were associated with stress-related disorders using animal models, especially focusing on stress susceptibility and resilience [51,52,53]. It was suggested that miR-15a-5p, let-7d-5p, miR-511-5p, and miR497a-5p, which target the two key post-traumatic stress disorder-related genes, *FKBP5* and *BDNF*, were differentially modulated in the several brain regions of the post-traumatic stress disorder-related susceptible and resilient mice [51]. Another study reported that 14 miRNAs, including let-7e, were modulated in the prefrontal cortex of three mouse strains, showing various susceptibilities to stress, subjected to the restraint stress paradigm [52]. More recently, it was suggested that the modulation of miR-144-3p could reduce the depression-related phenotypes in stress-susceptible mice [53]. These recent results strongly suggested that miRNA expression profiles could be reflected in the stress susceptibilities. Therefore, further studies using saliva from the multiple stress-susceptible and stress-resilient animals could provide diagnostic markers for identifying stress susceptibilities.

In the metabolome analyses, there were no salivary metabolites, which were significantly different between the sCSDS and control mice, in contrast to the present salivary miRNA profiles (Table 2). The differences between the present results obtained from miRNA-seq and metabolomic analyses might be derived from exosomes, which protect miRNAs from digestion [4].

This is the first report showing the potential candidates of salivary miRNA biomarkers of stress. Therefore, there are a few limitations in this study. First, to identify the robust and comprehensive salivary miRNA biomarkers, this study used saliva samples from the stress-susceptible mice only. However, further studies are needed using saliva samples not only from stress-susceptible mice but also from stress-resilient mice, as well as other stress model animals and humans to obtain accurate results. Second, we tested only male sCSDS and control mice. Therefore, the saliva samples from female depression models should also be analyzed in the future study [54].

## 4. Materials and Methods

### 4.1. Animals

This study was carried out according to the Law Concerning the Human Care and Control of Animals (Law No. 105; 1 October 1973), the Japanese Government Notification on the Feeding and Safekeeping of Animals (Notification No. 6; 27 March 1980), and the ARRIVE guidelines. It was approved by the Committee for Laboratory Animal Care and Use at Ibaraki University according to the guidelines of the Experimental Animal Committee (approval no.: 20170).

Male c57BL/6J (B6) (6-week-old) and Slc:ICR (ICR) mice (retired, older than 5 months) were obtained from CLEA Japan (Tokyo, Japan) and SLC (SLC Inc., Shizuoka, Japan), respectively. They were introduced into an air-conditioned room at Ibaraki University, and individually housed in a single cage (143 mm × 293 mm × 148 mm; Charles River Laboratories Japan, Kanagawa, Japan) with wood chips. All animals were given food and water ad libitum and were kept in our animal facility with a 12-h light/dark (lights on at 7:00 am). B6 mice were fed AIN-93G pellet chow (Oriental Yeast, Tokyo, Japan) and ICR mice were fed standard laboratory pellet chow (MF; Oriental Yeast).

### 4.2. Subchronic and Mild Social Defeat Stress (sCSDS)

The sCSDS paradigm was performed as previously described [22]. This paradigm consists of (1) selection of aggressive ICR mice, (2) short-term physical contact between ICR mice and B6 mice, and (3) long-term sensory interaction between them in a shared cage separated by a transparent device [22,23]. Importantly, the duration of physical contact was set at 5 min after the first attack bite on day 1, and was then reduced by 0.5 min per day from day 2 to day 10, as shown in a previous report [22]. The schedule of the present study is presented in Figure 1a.

First, aggressive selection of ICR mice was performed using a method similar to that described previously [22]. After 3 days of screening (three trials per day), aggressive behaviors of ICR mice were evaluated by their total length of time for attacking behaviors of each mouse, and 14 aggressive ICR mice were selected from 32 ICR mice. The retired B6 mice, which were not used for other experiments in this study, were used as screeners for the aggressive ICR mice selection. The selected aggressive ICR mice were transferred from single cages to individual social defeat (SD) cage (cage size: 220 mm × 320 mm × 135 mm; Natsume Seisakusho, Tokyo, Japan), which was divided into two compartments.

The B6 mice were divided into two groups: the sCSDS (*n* = 15) and non-stressed control (*n* = 6) groups after habituation to the environment of our facility for 2 weeks (from day −13 to day 0). From day 1 to day 10, test individuals (B6: sCSDS group) were exposed to a different ICR aggressor mouse each day at 10:00 a.m. To confirm the aggressive behaviors of all trials for 10 days of sCSDS sessions, their interactions were recorded with a video camera (Everio, JVC KENWOOD, Kanagawa, Japan). Aggressive behaviors were evaluated as described previously [55].

After physical contact, test mice were moved into the neighboring compartment to another aggressor for 24 h. As these two mice (B6 mice and aggressor ICR mice) were separated by the divider, there was no physical contact between them. However, psychological stressors from the aggressors, including visual, auditory, and olfactory stimuli, affected B6 mice. In each experiment, 15 B6 and 14 ICR mice were used for rotation. Control mice (B6) were kept in pairs in each compartment in SD cages with the divider for 10 days. The B6 mice were moved into the other compartment of the SD cage every day to change the combination of mice and environments, as well as the condition of the test mice. The sCSDS paradigm was performed twice for the microRNA-seq and metabolomic analyses. Therefore, we used a total of 42 B6 mice (30 in the stress group and 12 in the control group) in the present study.

### 4.3. Social Interaction Test

On day 11 in the morning (10:00 am), social behaviors were tested using previously described methods [19]. Briefly, a plastic interaction box with three wire-mesh windows was placed in an open-field arena (400 mm × 400 mm × 400 mm) and a 6–7 cm-wide area surrounding the interaction box was set as an interaction zone. Behaviors of the B6 mice were monitored for 2.5 min with the empty box (target absent), and subsequently the behaviors were monitored for 2.5 min in the target condition (with unfamiliar male ICR mice). SI scores (% of target absent) were calculated as 100 × (interaction time with target present/interaction time with target absent), as described previously [19]. A SI score >100 points indicates that the B6 mice exhibited socially interactive behavior with the unfamiliar male ICR mice. A SI score <100 points indicates that the B6 mice expressed lower socially interactive behavior with the ICR mice.

### 4.4. Sample Collection

After a 3-h fast in the morning (7:00 a.m.–10:00 a.m.) on day 12, the mice were anesthetized using 3% isoflurane for 3 min and intraperitoneally injected with 100 µL of 1.0 mg/mL pilocarpine-HCl (FujiFilm Wako Pure Chemical Corp., Osaka, Japan) to induce salivary secretion. Then, the mice were anesthetized using 3% isoflurane for 1 min, and saliva was collected using 200-µL micropipettes for 9 min. The saliva samples were centrifuged at approximately 5000× *g* for 15 min at 4 °C and stored at −80 °C until further use.

For the microRNA analysis, among the 21 B6 mice (6 and 15 mice in the control and sCSDS groups, respectively), five saliva samples from the control group and five from the sCSDS group with lower SI scores were selected and applied. For the metabolome analyses, among the 21 B6 mice, six saliva samples from the control group and six from the sCSDS group with lower SI scores were selected and applied.

### 4.5. Saliva microRNA-seq Analysis

Total RNA, including miRNA, was isolated from saliva samples using the MicroRNA Isolation Kit (BioChain Institute Inc., Newark, CA, USA). Total RNA samples were sent to the DNA Chip Research Inc., Tokyo, for the saliva microRNA-seq analysis. The quality of the RNA samples was assessed using an Agilent 2100 Bioanalyzer (Agilent Technologies, Santa Clara, CA, USA) with an Agilent RNA6000 pico kit and Agilent small RNA kit (Agilent Technologies). We confirmed that the samples consisted mostly of small RNAs. Sequencing libraries were constructed using the QIAseq miRNA Library Kit (Qiagen, Hilden, Germany). The QIAseq miRNA library kit adopts a unique molecular index (UMI) system, enabling unbiased and accurate quantification of mature miRNAs. The quality of the libraries was assessed using an Agilent 2100 Bioanalyzer (Agilent Technologies) with a high sensitivity DNA kit (Agilent Technologies). We confirmed that the library sizes were between 155 and 200 bp.

Equally pooled libraries were sequenced using NextSeq 500 (Illumina, Inc., San Diego, CA, USA) in 76-base-pair (bp) single-end reads. More than 10,000,000 reads were obtained from all the samples. The sequence reads were aligned to miRBase v21 (http://www.mirbase.org) and piRNABank version 1 (http://pirnabank.ibab.ac.in/) using the GeneGlobe data analysis center (Qiagen). All reads assigned to a particular miRNA or PIWI-interacting RNA (piRNA) were counted, and the associated UMIs were aggregated to count unique molecules. A matrix of the UMI counts of miRNA or piRNA was subjected to downstream analyses using Strand NGS 3.4 software (Agilent Technologies). UMI counts were quantified using the trimmed mean of the M-value method [56]. PCA was performed using the Strand NGS software (Strand Scientific Intelligence, Inc., San Francisco, CA, USA).

### 4.6. Gene Ontology and Pathway Analyses

Gene ontology analysis was performed using Strand NGS software. Pathway analysis was performed on the WikiPathways [57] database using the PathVisio tool [58] to determine pathways related to the genes associated with miRNAs that were significantly up/downregulated by sCSDS in saliva. Pathway analysis was conducted using miRNAs significantly up/downregulated (*p* < 0.05) and differentially expressed (fold change >2 or fold change <−2).

### 4.7. Saliva Metabolome Analysis (CE-FTMS)

The CE-FTMS was performed using an Agilent 7100 CE capillary electrophoresis system equipped with a Q Exactive Plus (Thermo Fisher Scientific Inc., Waltham, MA, USA), Agilent 1260 isocratic HPLC pump, Agilent G1603A CE-MS adapter kit, and Agilent G1607A CE-ESI-MS sprayer kit (Agilent Technologies). The CE-FTMS was conducted using an analyzer by Human Metabolome Technologies Inc. (HMT, Tsuruoka, Japan), according to HMT’s ω Scan package, using a previously described method with some modifications [59]. In short, 40 µL of saliva samples was added to 10 µL of Milli-Q water containing internal standards (H3304-1002, Human Metabolome Technologies Inc.). The solutions were filtered using a Millipore 5-kDa cutoff filter (ULTRAFREE MC PLHCC, Human Metabolome Technology Inc.) at 9100× *g* for 60 min at 4 °C. Then, the filtrates were employed for CE-FTMS analysis. PCA was performed using the HMT’s proprietary software, SampleStat.

### 4.8. Saliva Metabolome Analysis (LC-TOFMS)

Liquid chromatography time-of-flight mass spectrometry (LC-TOFMS) was carried out using an Agilent 1200 HPLC pump with an Agilent 6210 time-of-flight mass spectrometer (Agilent Technologies). LC-TOFMS was conducted using an analyzer by HMT Inc., according to HMT’s LC package, by performing previously described methods with some modifications [60,61]. Briefly, 80 µL of saliva samples was mixed with 240 µL of methanol containing internal standards (H3304-1002, HMT Inc., Tsuruoka, Japan). The mixtures were centrifuged at 2300× *g* at 4 °C for 5 min, and the supernatants were collected and evaporated. Then, the supernatants, resuspended in 160 µL of 50% isopropanol (*v*/*v*), were applied for LC-TOFMS analysis. PCA was performed using SampleStat software.

### 4.9. Additional Study for Detection of Histological Abnormalities

Some inflammation-related pathways were changed in sCSDS mice; we performed an additional study for the detection of histological abnormalities in the sCSDS mice organs. Male B6 mice (7-week-old at experiment initiation) were subjected to the sCSDS paradigm, using the method described above (four control and seven sCSDS mice). The mice were habituated to the environment of our facility for 1 week, and 14 aggressive ICR mice were selected from 21 ICR mice using the aforementioned method. After a 3-h fast in the morning (7:00 a.m.–10:00 a.m.) on day 11, the B6 mice were anesthetized using isoflurane. The mice were euthanized by blood collection from the inferior vena cava, followed by decapitation. In addition, male B6 mice (7-week-old at experiment initiation) were also subjected to the sCSDS paradigm (six control and six sCSDS mice). After a 3-h fast in the morning (7:00 a.m.–10:00 a.m.) on day 12 after the SI test, the mice were euthanized by cervical dislocation followed by decapitation. The hearts were then collected, immediately immersed in 10% neutral-buffered formalin, and embedded in paraffin wax. Sections were cut at 4-μm thickness and stained with hematoxylin and eosin (H&E). Tissue abnormalities were observed under a light microscope (Olympus, BX51, Tokyo, Japan) by a veterinary pathologist in a blinded manner. Quantitative analyses were performed using 10 continuous sections per heart at 4–6 µm thickness. The sections were stained with H&E and observed under a light microscope (Olympus BX50). The fibrotic areas (µm^2^) were analyzed using the Motic Images Plus 2.3S software (version 2.3.2; Motic Corp., Kowloon City, Kowloon, Hong Kong).

### 4.10. Statistical Analysis

Body weight, food intake, and water intake were analyzed by two-way ANOVA for repeated measures to test the factors of stress, time, and stress × time. SI scores were tested using Student’s unpaired *t*-tests. Data are shown as means ± SE. For miRNA-seq and metabolomic analyses, Welch’s *t*-test was used to compare stress factors. To control the *p*-value for multiple comparisons, the false discovery rate was determined using the method of Benjamini and Hochberg [62] as well as that of Storey and Tibshirani [63]. The significance threshold was set to *q*-value <0.1. In the pathway analysis, the pathways were considered significantly related to sCSDS, when the standardized difference score (*Z* score) was >1.96 and the permuted *p*-value was <0.05. The quantitative analyses of fibrotic areas of the hearts were analyzed by the unpaired *t*-test.

## 5. Conclusions

We characterized the miRNAs/piRNA and metabolic profiles modulated in the saliva of sCSDS mice. They could be potential candidates of early stress biomarkers in the saliva. In addition, these miRNAs might play important roles in the pathophysiological mechanisms of sCSDS, such as cardiac systems, adipogenesis, and oxidative stress responses. In the future, studies on the newly identified biomarkers in stressed human saliva will enable the development of reliable and early diagnosis of stress using saliva.

## Figures and Tables

**Table 1:** The effects of sCSDS on body weight, food intake and water intake determined by two-way ANOVA (control mice; *n* = 11, sCSDS mice; *n* = 11).

	Stress	Time	Stress × Time
Body weight	*F*_1,180_ = 3.28 *p* = 0.08	*F*_9,180_ = 18.42 *p* < 0.001	*F*_9,180_ = 0.97 *p* > 0.1
Food intake	*F*_1,180_ = 21.30 *p* < 0.001	*F*_9,180_ = 5.85 *p* < 0.001	*F*_9,180_ = 0.89 *p* > 0.1
Water intake	*F*_1,180_ = 59.05 *p* < 0.001	*F*_9,180_ = 5.00 *p* < 0.001	*F*_9,180_ = 2.89 *p* < 0.01

**Table 2:** The list of salivary miRNAs/piRNA significantly up/downregulated by sCSDS.

miRNA/piRNA	Fold Change	*p*-Value	*q*-Value
mmu-miR-6985-3p	7.66	5.36 × 10^−5^	0.0609
mmu-miR-7092-5p	9.13	9.25 × 10^−5^	0.0609
mmu-miR-208b-3p	10.77	9.34 × 10^−5^	0.0609
mmu-miR-378a-5p	12.21	0.000147	0.0685
mmu-miR-6944-3p	6.2	0.000175	0.0685
mmu_piR_000159	5.94	0.000217	0.0707
mmu-miR-3106-3p	−9.12	0.000332	0.0926
mmu-miR-3064-3p	7.33	0.000402	0.098

**Table 3:** The list of pathways significantly related to the miRNA-target genes in the saliva of sCSDS mice (*Z* score > 1.96 and permuted *p* < 0.05).

Pathway	Criterion for Z Score	Permuted *p*-Value
Oxidative phosphorylation	2.7	0.008
ApoE and miR-146 in inflammation and atherosclerosis	2.55	0.014
Small ligand GPCRs	3.34	0.016
BMP signaling pathway in eyelid development	3.12	0.021
Pentose phosphate pathway	2.77	0.023
Robo4 and VEGF signaling pathways crosstalk	3.04	0.033
Monoamine GPCRs	2.15	0.033

**Table 4:** The pathological phenotypes of the hearts in the control and sCSDS mice.

Mouse	Pathological Phenotype	Mouse	Pathological Phenotype
Control#1	-	sCSDS#1	Fibrotic tissue accumulation
Control#2	-	sCSDS#2	Fibrotic tissue accumulation
Control#3	Fibrotic tissue accumulation	sCSDS#3	Fibrotic tissue accumulation Inflammatory cell infiltration
Control#4	-	sCSDS#4	Fibrotic tissue accumulation
Control#5	Fibrotic tissue accumulation	sCSDS#5	Fibrotic tissue accumulation
Control#6	-	sCSDS#6	-
Control#7	-	sCSDS#7	Fibrotic tissue accumulation
Control#8	-	sCSDS#8	Fibrotic tissue accumulation
Control#9	-	sCSDS#9	Inflammatory cell infiltration
Control#10	-	sCSDS#10	-
		sCSDS#11	-
		sCSDS#12	-
		sCSDS#13	Fibrotic tissue accumulation

-: No pathological abnormality.

## Data Availability

Not applicable.

## Supplementary Materials

The following supporting information can be downloaded at: https://www.mdpi.com/article/10.3390/ijms232214479/s1, Figure S1a–w: The histological sections of the hearts from the control#1 (a), control#2 (b), control#3 (c), control#4 (d), control#5 (e), control#6 (f), control#7 (g), control#8 (h), control#9 (i), and control#10 (j) mice and sCSDS#1 (k), sCSDS#2 (l), sCSDS#3 (m), sCSDS#4 (n), sCSDS#5 (o), sCSDS#6 (p), sCSDS#7 (q), sCSDS#8 (r), sCSDS#9 (s), sCSDS#10 (t), sCSDS#11 (u), sCSDS#12 (v), and sCSDS#13 (w) mice. White arrow in (M and S) shows the area of inflammatory cell accumulation; Table S1: List of salivary miRNAs upregulated by sCSDS (fold change > 2).; Table S2: List of salivary miRNAs downregulated by sCSDS (Fold change < −2).; Table S3: The list of pathways related to the salivary miRNAs changed in sCSDS mice.; Table S4: The list of salivary metabolites up/downregulated by sCSDS measured by the CE-FTMS analysis (*p*-value < 0.05).; Table S5: The list of salivary metabolites up/downregulated by sCSDS measured by the LC-TOFMS analysis (*p*-value < 0.05).
