# Supplementary material for: Salivary microRNA and Metabolic Profiles in a Mouse Model of Subchronic and Mild Social Defeat Stress
Source: Int J Mol Sci. 2022 Nov 21;23(22):14479. doi: 10.3390/ijms232214479 (PMC9692636; doi:10.3390/ijms232214479)
Supplement: Supplementary file 1 [file ijms-23-14479-s001.zip › Supplementary Figure S1.pdf]

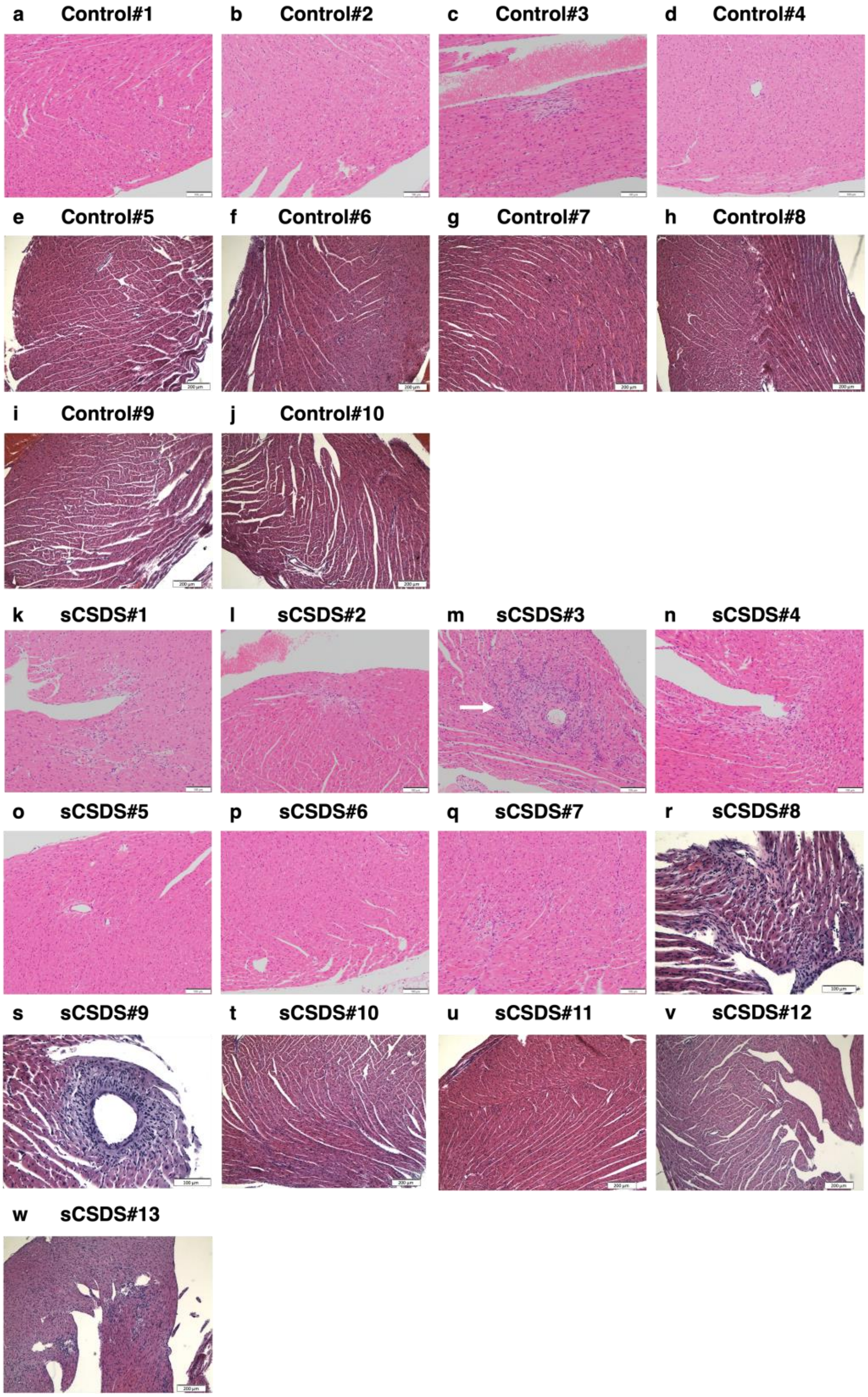

Supplementary Figure S1. a–w: The histological sections of the hearts from the control#1 (**a**), control#2 (**b**), control#3 (**c**), control#4 (**d**), control#5 (**e**), control#6 (**f**), control#7 (**g**), control#8 (**h**), control#9 (**i**), and

control#10 (**j**) mice and sCSDS#1 (**k**), sCSDS#2 (**l**), sCSDS#3 (**m**), sCSDS#4 (**n**), sCSDS#5 (**o**), sCSDS#6 (**p**), sCSDS#7 (**q**), sCSDS#8 (**r**), sCSDS#9 (**s**), sCSDS#10 (**t**), sCSDS#11 (**u**), sCSDS#12 (**v**), and sCSDS#13 (**w**) mice. White arrow in (M and S) shows the area of inflammatory cell accumulation.
